# Outcomes After Accelerated Partial Breast Irradiation in Women With Triple Negative Subtype and Other “High Risk” Variables Categorized as Cautionary in The ASTRO Guidelines

**DOI:** 10.3389/fonc.2021.617439

**Published:** 2021-03-11

**Authors:** Anabel Goulding, Lina Asmar, Yunfei Wang, Shannon Tole, Lora Barke, Jodi Widner, Charles Leonard

**Affiliations:** ^1^ Radiation Oncology, Rocky Mountain Cancer Centers, Denver, CO, United States; ^2^ Statistics, Linasmar Consulting, Houston, TX, United States; ^3^ Radiology, Invision Sally Jobe, Greenwood Village, CO, United States; ^4^ Surgery, SurgOne, Greenwood Village, CO, United States

**Keywords:** young age group, infiltrating lobular breast cancer, HER2 breast cancer +, estrogen receptor negative breast cancer, triple negative breast cancer, partial breast external beam radiotherapy

## Abstract

**Purpose:**

To report a primary objective clinical outcome of ipsilateral breast recurrence following accelerated partial breast irradiation (APBI) in women with triple negative and other high risk breast cancer (as described in 2017 ASTRO guidelines) (i.e., age 40–49, size 2.1–3.0 cm, estrogen receptor negative and invasive lobular breast cancer). Secondary objectives of axillary and regional failure as well as overall survival are also reported.

**Methods and Material:**

Patients from two clinical trials (NCT01185145, NCT01185132) were treated with 38.5 Gy IMRT or 3D-CRT APBI w/3.85 Gy fraction/BID fractionation for 10 fractions. Triple negative and other high risk patients (n=269) were compared to a total of 478 low risk patients which ASTRO defined as “suitable” for APBI. High risk patients, for the purpose of this study, were defined as those who possess one or more high risk criteria: triple negative (n=30), tumor size >2 cm <3 cm (n=50), HER 2+ (n=54), age range 40–50 years (n=120), ER- (n=43), and ILC histology (n=52).

**Results:**

Median follow up was 4.0 years for all patients. No significant difference was found for this high-risk cohort at 5 years for ipsilateral breast, or regional recurrences. Axillary recurrence was significantly adversely impacted by triple negative and ER- statuses (p=0.01, p=0.04). There were significant correlations between triple negative type and axillary recurrence on multivariate analysis (p=0.03). Overall survival for all patients was unaffected by any of the high-risk categories.

**Conclusion:**

The data from this study suggests that women possessing high risk features are at no more meaningful risk for recurrence than other patients considered to be acceptable for APBI treatment. However, the finding of axillary recurrence in patients with triple negative breast cancer does warrant a degree of caution in proceeding with accelerated partial breast irradiation technique in this patient group.

## Introduction

Accelerated partial breast radiotherapy (APBI) recently has been widely accepted as an alternative breast radiotherapy option for the post-lumpectomy adjuvant management of breast cancer. APBI has the benefit of shortened treatment time and reduced radiation exposure to surrounding tissues when compared to whole breast irradiation (WBI).

Contemporary external beam and brachytherapy APBI reports, including those of the authors, have reported that local control rates in certain early-stage invasive breast cancer patients may be comparable to those treated with standard whole breast (1–4). Optimal treatment outcomes of APBI are contingent upon proper patient selection.

The American Society of Radiation Oncology (ASTRO) has previously issued guidelines for patient categorization into “suitable”, “cautionary”, and “unsuitable” groups (2). Currently, these guidelines were revised to expand the suitable category to include characteristics previously felt to be cautionary (3). The GEC-ESTRO Brachytherapy Committee have also published recommended APBI clinical guidelines. These guidelines state that APBI could be offered as standard therapy to eligible patients >50 years of age who have T1 invasive ductal carcinoma with a minimum of 2 mm margins (4). The National Comprehensive Cancer Network (NCCN) panel accepts the updated 2016 version of the ASTRO APBI guideline, which now defines patients “suitable” for APBI to be the following: 1) 50 years or older with invasive ductal carcinoma (IDCA) measuring ≤2 cm (T1 disease) with negative margin widths of ≥2 mm, no lymphvascular invasion, estrogen receptor (ER) positive, and BRCA 1/2 negative or 2) screening-detected ductal carcinoma *in situ* (DCIS) with low/intermediate nuclear grade, and tumor size measuring ≤2.5 cm with negative margin widths of ≥3 mm (1).

The cautionary group of patient characteristics now includes: age 40–49, size of 2.1–3 cm, estrogen receptor negative, and invasive lobular histology (according to ASTRO). There have been only a few reports which document the APBI experience with this cautionary subgroup of patients and these pertain nearly exclusively to brachytherapy techniques (5–13).

This is a retrospective analysis of a total of 269 patients with high risk characteristics, including triple negative, who have been enrolled into two separate accelerated partial breast trials, prospective phase II (NCT01185145) and phase III (NCT01185132) clinical trials. Historically, reports have been divided in the outcomes of these patients. There are whole breast radiotherapy reports which state that the local/ipsilateral breast control in patients with triple negative breast cancer are significantly lower than patients without triple negative or basal type tumors (14–19). There were similar conclusions in young patients (20–31) and in patients with infiltrating lobular histologies (32–34) and HER2/neu positive cancers (15–17, 25, 35) which show higher recurrence rates than in older patients with non- lobular or HER2/neu positive tumors. In contrast, other reports utilizing external beam/brachytherapy irradiation have not observed any worse loco-regional recurrence outcomes in patients with triple negative/basal type of breast cancer (36–41), young age (42–44) or infiltrating lobular histologies (45–51) when compared to older patients with non- lobular or HER2/neu positive tumors.

## Methods

A total of 747 patients enrolled in two accelerated partial breast protocols were used in this analysis. Eligibility for both trials were very similar and included patients with clinically unifocal invasive breast cancer which measured up to 3 cm in size. Patient characteristics are in **Table 1** and protocol eligibility requirements including a minimum of ≥2 mm margins and treatment guidelines have been previously reported (52, 53). High risk patients, for the purpose of this study, were defined as those who possess one or more high risk criteria: triple negative (n=30), tumor size ≥2 cm ≤3 cm (n=50), HER2 + (n=54), age range 40-50 years (n=120), ER- (n=43), and ILC histology (n=52). Data collection did not include variables such as limited/focal lymph vascular invasion (LVI) or extensive intraductal component (EIC). **Table 2** also delineates an analysis of shared high risk characteristics. Clinical outcomes of ipsilateral breast, axillary, and combined regional recurrences (ipsilateral or axillary) (RR), and overall survival (OS) were analyzed and compared in each high-risk cohort.

**Table 1:** Patient characteristics.

Characteristic	All patients
Age at diagnosis (*y*), mean (SD)	62 average (11.0)
Median (range)	62 (37–96)
Menopausal status at study entry, *n (%)*	
Pre/Perimenopausal	148 (19.8%)
Postmenopausal	599 (80.2%)
Primary histology, *n (%)*	
Invasive ductal carcinoma	691 (92.5%)
Invasive lobular carcinoma	52 (7.0%)
Invasive mammary carcinoma	4 (0.5%)
Margin size (cm)	
Median (Range)	0.7 (0–3.0)
Estrogen receptor status (*n*)	
Positive	702 (93.4%)
Negative	43 (5.8%)
Unknown	2 (0.2%)
HER2/neu status (*n*)	
Positive	54 (7.2%)
Negative	683 (91.4%)
Unknown	10 (1.3%)
ER negative and HER2 negative (*n*)	32 (4.3%)
T stage (*n*)	
T1mic	14 (1.9%)
T1a	92 (12.3%)
T1b	332(44.4%)
T1c	279 (37.4)
T2	30 (4%)
N stage (*n*)	
N0	728 (97.5%)
N0(i+)	19 (2.5%)
Bilateral breast MRI prior to enrollment (*n*)	632 (87.1%)

**Table 2:** Actuarial 5-year overall survival, ipsilateral breast recurrence-free survival (RFS), axillary RFS, and regional (breast and axillary) RFS.

VARIABLE		5-year OS (%)	p	IPSILATERAL BREAST RFS (%)	p	AXILLARY RFS (%)	p	REGIONAL RFS (%)	p
HISTOLOGY	ILCA	88.9	0.14	100	0.45	97.5	0.07	97.5	0.75
	IDCA/IMC	95.8		98		99.7		97.7	
AGE	< 50	96.6	0.1	97	0.28	100	0.45	97	0.55
	>50	95.2		98.3		99.5		97.8	
TRIPLE NEGATIVE	YES	84.3	0.16	100	0.34	96.7	0.01	96.7	0.93
	NO	95.9		97.9		99.7		97.6	
HER2/NEU	NEGATIVE	95.3	0.88	97.9	0.45	99.5	0.64	97.4	0.38
	POSITIVE	95.8		100		100		100	
SIZE	<2 cm	95.9	0.11	98.2	0.56	99.5	0.66	97.7	0.77
	≥2 cm	87.4		95.8		100		95.8	
ER	NEGATIVE	87.5	0.29	100	0.3	97.5	0.04	97.5	0.94
	POSITIVE	96		97.9		99.7		97.6	

### Statistical Analyses

Continuous variables were presented as mean with standard deviation and median with ranges. Categorical variables were expressed as counts with percentages. Kaplan-Meier method with log-rank test was used to estimate the overall survival and the recurrence-free survivals. Univariate and multivariable Cox regression models, which including variables of age, histology, tumor size, and hormone receptor status, were performed to evaluate risk factors associated with death and recurrences.

In addition to the main analysis, we performed a sub-analysis matching the recurrent patients with non-recurrent ones. Variables used for matching were age, histology, tumor size and hormone receptor status. For each sub-analysis, we matched variables for patients with and without recurrences and analyzed one risk factor which was not matched. SAS version 9.4 (SAS Institute, Cary, NC, USA) was used for all statistical analyses.

## Results

There were 269 patients in the high-risk study group which also includes 30 patients with triple negative subtype breast cancer. High-risk/triple negative patients were compared against a total of 478 patients. Median follow up was 4.0 years for all patients. Of all high- risk patients/triple negative, 70 patients had two or more high-risk characteristics. **Table 3** shows that no significant overall survival, ipsilateral breast or regional relapse-free survival differences were found for this high-risk cohort at 5 years as compared to low risk patients. There were also no significant differences for ipsilateral breast, axillary or regional (ipsilateral breast *or* axillary) recurrences in the infiltrating lobular, age ≤50, HER2/neu positive or tumor size ≥2 cm between cohorts. However, the triple negative subtype was found to significantly adversely impact axillary recurrence. Other “high risk” variables such as the ER negative subtypes were also found to significantly adversely impact axillary recurrence.

**Table 3:** Univariate analysis.

VARIABLE		HR (95% CI) for 5-year survival	p	HR (95% CI) for IPSILATERAL BREAST recurrence	p	HR (95% CI) for AXILLARY RECURRENCE	p	HR (95% CI) for REGIONAL RECURRENCE	p
HISTOLOGY	ILCA v IDCA/IMC	2.156 (0.756, 6.147)	0.15	N/A*	N/A*	6.88 (0.62,75.84)	0.12	1.39 (0.18,10.78)	0.75
AGE	≤ 50 v >50	0.315 (0.075, 1.319)	0.11	2.07 (0.53, 8.10)	0.3	N/A*	N/A*	1.47 (0.40, 5.40)	0.56
TRIPLE NEGATIVE	YES v NO	2.137 (0.726, 6.286)	0.17	N/A*	N/A*	10.95 (0.99,120.8)	0.05	1.10 (0.13, 9.17)	0.93
HER2/NEU	NEGATIVE v POSITIVE	1.120 (0.267, 4.702)	0.88	N/A*	N/A*	N/A*	N/A*	N/A*	N/A*
SIZE	<2 cm v ≥2 cm	2.300 (0.806, 6.559)	0.12	1.83 (0.23,14.56)	0.6	N/A*	N/A*	1.36 (0.18,10.47)	0.77
ER	NEGATIVE v POSITIVE	0.567 (0.194, 1.653)	0.3	N/A*	N/A*	0.12 (0.01, 1.36)	0.08	1.09 (0.13, 8.84)	0.94

*Due to a lack of any events p-values are not generated.

On univariate analysis, triple negative status was also associated with decreased axillary recurrence-free survival (p=0.051) (**Table 3**). The multivariate analysis in **Table 4** depicts the only significant correlations which were between triple negative type and decreased axillary recurrence-free survival (p=0.03).

**Table 4:** Multivariate analysis.

VARIABLE		HR (95% CI) for 5-year survival	p	HR (95% CI) for IPSILATERAL BREAST recurrence	p	HR (95% CI) for AXILLARY RECURRENCE	p	HR (95% CI) for REGIONAL RECURRENCE	p
HISTOLOGY	ILCA v IDCA/IMC	2.25 (0.78, 6.47)	0.13	N/A*	N/A*	13.09 (0.82,209.3)	0.07	1.48 (0.19,11.62)	0.71
AGE	< 50 v >50	0.33 (0.08, 1.38)	0.13	2.07 (0.53, 8.10)	0.26	N/A*	N/A*	1.53 (0.41, 5.71)	0.52
TRIPLE NEGATIVE	YES v NO	2.38 (0.78, 7.22)	0.13	N/A*	N/A*	20.35 (1.27,325.4)	0.03	1.07 (0.13, 9.13)	0.95
SIZE	<2 cm v ≥2 cm	1.87 (0.65, 5.43)	0.25	1.83 (0.23,14.56)	0.46	N/A*	N/A*	1.43 (0.18,11.31)	0.74

*Due to a lack of any events p-values are not generated.

### Matched Pair Analysis

The matched pair analysis is shown in **Table 5**. The only significant difference between the high and low risk APBI cohorts was for axillary recurrence free survival and overall survival for ER – patients (p=0.03, p=0.013). There were no significant differences in the remaining high risk cohorts for overall survival, ipsilateral breast recurrence-free, axillary recurrence- free, or regional recurrence-free survival outcomes.

**Table 5:** Matched pair analysis.

VARIABLE	MATCHED PAIR	OS p-value	Ipsilateral Breast RFS p-value	Axillary RFS p-value	REGIONAL RFS p-value
ER -	1:5	0.01	0.37	0.03	0.81
HER2/neu	1:5	0.62	0.41	N/A*	0.41
LOBULAR HISTOLOGY	1:5	0.15	0.37	0.2	0.99
TRIPLE NEGATIVE	1:5	0.57	0.15	0.24	0.54
SIZE	1:5	0.33	0.16	N/A*	0.16
AGE	1:5	0.17	0.21	0.54	0.38

*Due to a lack of any events p-values are not generated.

## Discussion

The current guidelines from various organizations are not firmly based on APBI data which document that ipsilateral breast tumor recurrences (IBTR) are higher among certain subsets of patients including those with triple negative tumors. Rather, these groupings represent a conservative approach to patient APBI eligibility due to available contradicting data. The authors do recognize that these guidelines are for the use of APBI outside of clinical trial and are updated to reflect new research findings to provide continuing direction for the use of APBI. One can even find a lack of consistency between the ASTRO and GESTRO consensus guideline statements, including tumor size and estrogen receptor status (2, 4).

As well, the publication of other reports would suggest that the standard use of APBI might extend beyond the scope of these recommended patient groups.

Current reports have been relatively inconsistent in identifying particular variables which may impact ipsilateral breast tumor recurrence and have had inconsistent findings in other aspects of regional/distant control. As discussed previously, accelerated partial breast radiotherapy can be administered with brachytherapy as well as external beam radiotherapy (3-dimensional, intensity modulated and proton techniques). Several reports document the APBI brachytherapy experience with patients who are categorized in the “cautionary” and/or “unsuitable” poor prognostic variables (5–13). A combined Mammosite Registry and William Beaumont experience with partial breast brachytherapy reported that there were no significant differences in ipsilateral breast failures in the unsuitable cohort versus the “suitable” or “cautionary” cohorts (4.6% versus 2.5% and 3.3% respectively; p=0.2). However, age (<50 vs ≥50) as well as estrogen receptor status (negative versus positive) were significant factors for ipsilateral breast failures (7).

The University of Wisconsin published findings in patients with “high” risk/cautionary features (17, 18). On univariate analysis, both ER negative receptor status and lobular histology were significantly associated with ipsilateral breast failure (p= 0.002 and 0.0004, respectively). Multivariate analysis, however, failed to identify any cautionary feature associated with breast failure. William Beaumont Hospital did not find any significant differences in local breast failure across “suitable”, “cautionary”, or “unsuitable” subgroups in 199 APBI patients when compared to a matched cohort of 199 whole breast patients after a median follow-up of 9 and 13 years for the two groups respectively (10). Univariate analysis of APBI patients did not result in any variable which was significantly associated with ipsilateral breast recurrence. However, as noted in our study, regional nodal failure was significantly associated with ER negative receptor status and positive nodal status in the APBI cohort.

Several other accelerated partial breast irradiation reports found that negative estrogen receptor status could result in a higher ipsilateral breast recurrence and/or distant failure (6, 12, 13).

Studies examining the efficacy of WBI on high risk patients have reported similarly inconsistent results as APBI studies in identifying suitable characteristics for treatment (14, 15, 26, 28, 34, 36, 43, 44). Just as in the case of APBI data, these WBI studies have had equivocal conclusions and, as a whole, have not consistently agreed on all exclusion/inclusion criteria for APBI patients.

While continued, supporting data is needed, the comparability in study outcomes of APBI vs WBI treatment suggests that high risk patients are at no more meaningful risk for recurrence when treated with APBI than WBI. The data reported here as well the other studies cited above suggest that APBI might also be used as a standard of care treatment for the cautionary group analyzed in this study.

The larger phase III trials which randomized APBI versus WBI have had varying but similar eligibility criteria (54–59). Generally, these trials have included patients age ≥40 except RTOG 0413 which included patients ≥18 and IMPORT-LOW which only allowed patients ≥50. None of these specifically excluded ER negative patients, HER2/neu positive patients and the Import Low and RAPID trials disallowed invasive lobular. However, infiltrating ductal comprised greater than 85% of the patient populations of these studies with RTOG 0413 stating 4% of their APBI cohort was infiltrating lobular. None of these studies disallowed ER negative patients but this population only was approximately 5%–8% (RTOG 0413 had 19% ER/PR negative patients) of their APBI cohort. Tumor size for the RAPID, IMPORT-LOW, and RTOG 0413 was ≤3 cm and was 2.5 cm and 2 cm for the Florence and Hungarian trials respectively. Although HER2/neu positivity was not considered to an exclusion criterium in these trials, it has only been reported in the Florence (2.8%) and IMPORT-LOW trials (4%). At this time, however, there have been no data from these phase III studies which have driven any consensus toward definitive data- driven conclusions.

Limitations for this study include the sample size and the length of follow-up. Of all 747 patients that were enrolled in our clinical trials, 269 patients were defined as high risk for the purpose of this study. To our knowledge this is the largest study analyzing the use of APBI in high risk women. Further studies with increased sample sizes are needed for corroboration of the results presented. The median follow-up for this study is 4.0 years. Prior reports have shown that median times to ipsilateral breast relapse in patients with ASTRO defined cautionary characteristics such as triple negative, estrogen receptor negative and HER2/neu positive range from 3–4 years (32, 34, 60). Other studies have also reported median disease-free intervals of 2–3 years in this category (19, 61–63).

## Conclusion

The data presented in this study shows that there should be continued reconsideration for inclusion of at least several high-risk variables such as estrogen receptor negative, triple negative, HER2/neu positive,/2–3 cm primary tumors, age 40–50 patients, and patients with infiltrating lobular tumors. Age, histology, and tumor size do not appear to affect favorable outcomes. However, although there is evidence to suggest that there should be continued caution for APBI patient selection of triple negative and estrogen receptor negative tumors, these differences may not be of any meaningful clinical differences whether WBI or APBI is utilized. Further studies and/or follow-up must be done to further corroborate whether these patients, especially those with triple negative disease, should be included or not as eligible for APBI.

## Data Availability Statement

The raw data supporting the conclusions of this article will be made available by the authors, without undue reservation.

## Ethics Statement

The studies involving human participants were reviewed and approved by Western IRB. The patients/participants provided their written informed consent to participate in this study.

## Author Contributions

LA and YW provided statistical analysis and manuscript writing. AG, ST, and CL contributed to the data analysis and manuscript preparation. LB and JW contributed to the manuscript preparation. All authors contributed to the article and approved the submitted version.

## Conflict of Interest

LA and ST were employed by Linasmar Consulting.

The remaining authors declare that the research was conducted in the absence of any commercial or financial relationships that could be construed as a potential conflict of interest.
